# Inequities in food access during the COVID-19 pandemic: A multilevel, mixed methods pilot study

**DOI:** 10.1186/s12889-025-25964-3

**Published:** 2026-01-14

**Authors:** Megha R. Aepala, Alice Guan, Tessa Cruz, Jamaica Sowell, Brenda Mathias, Katherine Lin, Analena Hope Hassberg, Salma Shariff-Marco, Mindy C. DeRouen, Antwi Akom

**Affiliations:** 1 Department of Epidemiology and Biostatistics, University of California, San Francisco, CA USA; 2 The Social Innovation and Urban Opportunity Lab, Streetwyze, UCSF & San Francisco State University, Oakland, CA USA; 3 Roots Community Health Center, Oakland, CA USA; 4 Department of Sociology, California State University, Los Angeles, CA USA; 5 Department of Africana Studies, San Francisco State University, San Francisco, CA USA

**Keywords:** Food insecurity, Mixed methods, Community-based participatory research, Mobile health technology

## Abstract

**Background:**

Innovative data integration may serve to inform rapid, local responses to community needs. We conducted a mixed methods pilot study among communities of color or low-income in the San Francisco Bay Area amid the COVID-19 pandemic to assess a hypothesized data model to inform rapid response efforts.

**Methods:**

Between 2020–2021, we collected (1) qualitative data through neighborhood reports submitted via Streetwyze, a mobile neighborhood mapping platform; (2) survey data on social and economic circumstances; and (3) geospatial data among residents of three counties. Qualitative data were coded and then integrated with survey and geospatial data. We used descriptive analyses to examine participants’ experiences with food in their neighborhoods.

**Results:**

Among 51 participants**,** seventy percent of participants reported food insecurity before and after the pandemic began in March 2020. Within neighborhood reports, *food* was the most frequently occurring sub-theme within the *Goods* and *Resources* parent themes (68% and 49% of reports, respectively). *Security* (88%), *resource programs* (88%), *outdoor space* (84%), and *equity* (83%) were more likely to be mentioned by participants who were food insecure compared to those who were not (12%, 12%, 16%, 17%, respectively). Mentions of food in neighborhood reports more often occurred in census tracts with lower socioeconomic status and more area-level food insecurity.

**Conclusion:**

Individuals who were food insecure reported a constellation of needs beyond food, including needs related to safety and greater social equity. Our data model illustrates the potential for rapid assessment of community residents’ experiences to provide enhanced understanding of community-level needs and effective support in the face of changing circumstances.

**Supplementary Information:**

The online version contains supplementary material available at 10.1186/s12889-025-25964-3.

## Introduction

Food insecurity and other neighborhood-level social determinants of health (SDOH) are shaped by complex, multilevel factors, including economic conditions, infrastructure, and community resources. These inequities deepened during the COVID-19 pandemic, which disproportionately affected racial/ethnic minority and low-income populations. Black and Hispanic households, already facing food insecurity rates more than double those of White households, encountered additional barriers such as job loss, transportation limitations, language barriers, and ineligibility for public assistance among some immigrant groups [1–4]. Despite emergency measures like expanded SNAP benefits, food insecurity remained persistently high among low-income Black and Hispanic adults [3], highlighting the need for interventions that address structural barriers and discrimination. Understanding these determinants requires methodologies that capture both lived experience and contextual factors at fine spatial and temporal scales.

Recent literature suggests that integrating qualitative and quantitative data with geospatial analysis offers a nuanced understanding of neighborhood-level health determinants. For example, Geographic Information Systems (GIS) are used to map food environments and link them to chronic disease risk, enabling visualization of disparities and identification of intervention targets [5]. Mobile ecological momentary assessment (EMA) platforms allow real-time, location-specific data collection on food deserts, pollution, or social stressors in relation to health behaviors and outcomes, increasing the granularity and contextual relevance of findings [6]. Mobile daily diaries, as piloted in environmental justice neighborhoods [7], enable repeated, participant-driven data collection on stressors and acute health symptoms, supporting integration of open-ended qualitative data with measured environmental exposures. Participatory mapping approaches, including qualitative GIS (QGIS), further extend these capabilities by integrating community perspectives into spatial analyses, addressing data gaps, and providing context-specific insights into food security and other health-related issues [8, 9]. Studies that integrate these various data types, however, would benefit from an explicit data framework and approach that maximizes knowledge to be gained. For example, many GIS-based studies rely on static, publicly available datasets that may not capture real-time changes in community needs, a limitation amplified during crises such as the COVID-19 pandemic.

Community engagement, particularly models with shared leadership where community members are equal partners in decision-making, enhances the equity, cultural relevance, and sustainability of public health interventions [10]. In food access initiatives, such approaches address multiple equity domains and produce more durable outcomes. Participatory methods like co-design and community food researcher models empower residents to identify priorities, guide solutions, and embed community perspectives throughout all research phases, strengthening trust, uptake, and long-term impact [11–13]. However, while community-engaged methods are well established, few studies integrate community-generated qualitative data, quantitative surveys, and geospatial analyses in a sustained, real-time framework.

Our study addresses these gaps through a convergent mixed-methods, community-based participatory research (MMCBPR) pilot conducted in Alameda, Contra Costa, and San Francisco counties during the COVID-19 pandemic [14]. We incorporated continued feedback on participants’ experiences within their neighborhoods through Streetwyze, a mobile mapping platform designed with and for communities of color or low-income [14]. During our study, we used emerging themes from these neighborhood reports to inform the development of repeated epidemiological surveys that assessed several domains of structural and social determinants of health (employment, healthcare access, housing, child/eldercare, transportation, food, finances, everyday discrimination) and well-being (COVID-19 stressors and physical/mental health). Recently, we also published an inductive analysis of neighborhood reports that describes three themes participants found particularly salient during the pandemic; innovation to foster community cohesion and establish informal networks; the value and importance of racial, ethnic, and culturally tailored services; and dignity in service [15]. In addition to neighborhood reports (qualitative data) and surveys (quantitative data), we collected multiple forms of geospatial data including participants’ residence, locations associated with neighborhood reports, locations of resources listed in public resource directories and secondary neighborhood data. To further leverage the three data types collected, we now describe a data model that integrates them and illustrates the potential utility of doing so in the context of food access among study participants. The purpose of illustrating this data model is to present a real-world application of how sustained community engagement via a mobile platform and data integration can be used to identify localized, targeted, and specific areas of intervention to address salient community needs and expand community-identified solutions to unmet needs.

## Methods

### Study design

During the COVID-19 pandemic, our study team comprised of researchers at the University of California San Francisco, San Francisco State University, Digital Organizing Power-Building and Engagement (DOPE Labs, which supports Streetwyze) and Roots Community Health Center (Roots) designed a mixed methods community-based participatory research (MMCBPR) study to understand the impacts of the pandemic on three San Francisco Bay Area counties. Complete details on the MMCBPR study design, including how mixed methods were integrated into each of the seven core phases of community-based participatory research, are described elsewhere [14]. The study was approved by the University of California, San Francisco Human Research Protection Program (IRB #20–31055) and the Institutional Review Board for San Francisco State University.

### Participant recruitment

Adults who spoke English or Spanish, had access to a digital device, and lived within San Francisco, Alameda, or Contra Costa counties were eligible to participate. We recruited a convenience sample of individuals through community-based organizational partners (Streetwyze, Roots), social media (Instagram, Twitter), printed advertisements (fliers, posters), and outreach presentations during existing virtual support group meetings. This approach was necessary given COVID-19 restrictions on in-person recruitment and the need to rapidly engage established community networks to ensure timely data collection during a period of heightened need.

We also invited existing Streetwyze users to participate. A total of 75 participants consented into the study; 51 (68%) completed the baseline quantitative survey, and 19 (25%) submitted qualitative neighborhood reports via Streetwyze. Eighteen of these 19 qualitative respondents also completed the baseline survey, making the qualitative sample largely a subset of the quantitative sample.

### Data collection

*Qualitative data *were collected as participant-submitted neighborhood reports in Streetwyze, a mobile mapping and SMS platform that allows for real-time mobile data collection via neighborhood reports, ratings, and reviews reflecting participants’ lived experiences [14, 15]. Within the Streetwyze platform, participants were directed to describe their experiences with resources and services during the pandemic through tailored focus questions (e.g., “Where is the safest and most affordable place for you to go to get the things you need?”, “Are there community-based organizations or neighbors who are making a difference in your life that you’d like to highlight?”). While participants were provided multiple format options to share their experiences (i.e., video, audio, or text), all reports we received in this study were text-based. A total of 236 neighborhood reports from 19 participants (range: 10 to 27 reports per participant) were received. Codebook development as well as formal inductive analysis of these reports have been published elsewhere [14, 15]. Briefly, the codebook was developed by a team of academic and community researchers to capture themes across neighborhood reports through preliminary assessment and discussion. A hierarchy of themes was developed to summarize six topical categories, which included: goods, resources, access, infrastructure, well-being, and infection control. Each of these “parent themes” included several sub-codes to reflect specific topics that were mentioned in the reports. For example, the *access *parent theme was subdivided into availability, convenience, price, orderliness, quality, security, and service (Supplemental Fig. 1). Each neighborhood report was independently coded by one community and one academic researcher; code conflicts were resolved by consensus among the full coding team.

We calculated the average number of mentions of each sub-code and parent theme. Then, each participant was assigned an indicator value to designate whether they mentioned each sub-code or parent theme more than average.

*Quantitative data* were collected through an epidemiological survey (*N* = 51) via REDCap [16, 17] between January 1, 2021-October 31, 2021 and assessed participants’ circumstances prior to, and following, the COVID-19 pandemic shelter-in-place orders in the San Francisco Bay Area (March 2020).

Survey domains included demographic characteristics (age, gender, race/ethnicity) along with social and structural determinants of health (employment, healthcare, housing, childcare, transportation, food, well-being, and financial circumstance). The survey instrument incorporated items from the PhenX Toolkit and other validated sources [18–35]; the full baseline and follow-up surveys are available as Supplemental Methods and include source references relevant to specific survey items. Validated measures included the Global Physical Health and Global Mental Health scales for overall health status, and the Everyday Discrimination Scale for discrimination experiences in the past year. Responses to the Everyday Discrimination Scale were additionally coded for chronicity following established methods [18]. In some cases, instruments were added (e.g., COVID-related safety, coping) or adapted (e.g., pandemic-related concerns) to our study based on integration of results of qualitative data analysis according to a convergent mixed methods approach [14].

Food insecurity was assessed by adapting the six-item standard measure from the U.S. Department of Agriculture Economic Research Service (Supplemental Methods) [34]. “Food Insecure Ever” was defined as reporting food insecurity in either the pre-pandemic (before March 2020) or pandemic (after March 2020) period, and “Persistent Food Insecurity” as reporting food insecurity in both periods.

### Geospatial data

All (100%) participants who completed the baseline survey provided a full residential address (*n *= 50) or cross-streets of residence (*n* = 1). Addresses and cross-streets were geocoded to latitude/longitude coordinates using Arc GIS [36]; census tract identifiers for 2010 Census geographies were then appended. The Census 2020 geographies were not yet available at the time of analysis.

Measures of census tract-level neighborhood SES (nSES) and percent food insecure were obtained from UCSF Health Atlas [37]. Neighborhood SES was previously created with principal components analysis of measures related to census tract-level income, occupation, education, and housing with data from the American Community Survey (2013–2017); quintiles are based on the distribution of index scores among all census tracts in the state of California [38]. The proportion of individuals within a census tract experiencing food insecurity was from Feeding America’s Map the Meal Gap study (2016 and 2017) [39]. Although these secondary data precede the pandemic-era primary data collection period, they were the most current tract-level nSES measures available in the Health Atlas at the time of analysis [37]. We use census tract–level data as a proxy for neighborhood-level measures, recognizing that census tract boundaries may not fully align with residents’ perceptions of their neighborhoods.

Neighborhood reports provided via the Streetwyze mapping platform are geocoded to latitude/longitude coordinates within the Streetwyze application and were then assigned to 2010 census tracts.

For this study, a “food resource” was defined as any emergency food assistance program (e.g., food banks, community-based meal distribution programs), government food assistance program (e.g., CalFresh, WIC, Pandemic Electronic Benefit Transfer), or free meal program (e.g., school meal programs). We did not include commercial food retail outlets or restaurants.

Presence of food resources was determined via Alameda, Contra Costa, and San Francisco county directories (collected March 2021); the address of each resource was geocoded using Google API [40]; census tract identifiers were then appended. The frequency of resources per capita was calculated for each census tract using population estimates from Census 2010 [41].

### Data analysis and integration

We use proportions or means and associated standard deviations to provide an overall description of sociodemographic characteristics of the study sample according food insecurity.

Each sub-code and parent theme were summarized by food insecurity status. We found that the compound effects of experiencing multiple forms of stressors can influence an individual’s wellbeing. Thus, sub-codes within the resources, well-being, and infastructure parent themes (parent themes that appeared to differ substantially in occurrence between those who were never and ever food insecure) were summarized by according to whether participants reported less or more than the average number of COVID-19 stressors.

We integrated geospatial measures with qualitative and quantitative data by (1) overlaying the locations where participants reported receiving food resources via survey atop a base map illustrating the proportion of food resources for each census tract and (2) illustrating locations where participants mentioned food resources in neighborhood reports atop census tract-level base maps illustrating nSES and proportion of residents who experienced food insecurity. All maps were created with ArcGIS using shapefiles for Census 2010 tract boundaries available from the National Historical Geographic Information System [36, 42, 43].

## Results

A total of 51 participants completed the baseline survey (Table 1). Most participants lived in Alameda County (85.7%), were between 18–45 years of age (77.6%), and were women (74.0%). Participants were racially/ethnically diverse: 43.1% identified as Black or African American, 29.4% as Hispanic or Latino, 19.6% as Asian, and 9.8% as White. Over half (56.9%) reported annual household incomes below $50,000 and more than two-thirds had less than 6 months of household savings (68.6%). Most participants reported changes in employment (68.6%) and transportation (60.8%) from before to after the pandemic began in March 2020. Participants reported experiencing an average of 7.7 ± 4.1 COVID-19–related stressors and an average of 202.5 ± 376.9 everyday experiences of discrimination, indicating notable pandemic-related stress and experiences of discrimination in the past year.

**Table 1:** Frequency distribution of sociodemographic characteristics of study participants according to self-reported food insecurity (Survey data)

	**Total participants** ***N* = 51**	**Food insecure ever** ***N* = 40**	**Persistent food insecurity** ***N* = 34**
	% or mean (standard deviation)		
Total		74.5	70.6
County			
Alameda	85.7	86.8	87.5
San Francisco/Contra Costa	14.3	13.2	12.5
Age group (in years)^1^			
18–45	77.6	81.6	84.4
46 +	22.5	18.4	15.6
Race or ethnicity^1, 2^			
Asian American	24.0	23.1	24.2
Black or African American	28.0	25.6	30.3
Hispanic	28.0	33.3	30.3
White and Other	20.0	18.0	15.2
Gender^1^			
Man	24.0	23.1	24.2
Woman	74.0	74.4	72.7
Household savings			
Less than a month	35.3	42.5	47.1
1–2 months	33.3	37.5	35.3
3–6 months	17.7	12.5	11.8
More than 6 months	13.7	7.5	5.9
Employment change^3^	68.6	75.0	79.4
Housing change^3^	49.0	55.0	55.9
Transportation change^3^	60.8	60.0	61.8
Healthcare change^3^	29.4	35.0	35.3
Number of COVID-19 stressors^4^	7.7 (4.1)	8.2 (4.1)	8.2 (4.1)
Good/very good/excellent physical health^5^	78.4	80.0	79.4
Good/very good/excellent mental health^5^	58.8	57.5	52.9
Number of past year discriminatory experiences	202.5 (376.9)	240.8 (414.4)	274.6 (441.2)

^1^Missing data: age (*n* = 1), race or ethnicity (*n* = 1), gender (*n* = 2)

^2^The “other” category of race or ethnicity includes Middle Eastern/North African and American Indian/Alaska Native. Financial circumstance was evaluated by asking participants how long they could maintain their lifestyle with their current savings

^3^Changes to employment, housing, transportation, and healthcare were assessed by asking participants if these factors change following pandemic restrictions in March 2020

^4^Participants selected from a list of 17 different possible COVID-19 stressors, including concerns for health of self, health of family members, financial concerns, impact on work, impact on child(ren), impact on community, impact on relationship with adult family members, access to food, access to baby supplies, access to personal care products or household supplies, access to healthcare, access to housing, ability to parent how I want, ability to care for older adults or people with disabilities, social distancing or being quarantined, transportation and safety, or something else [35].

^5^PROMIS Global Physical Health and Global Mental Health [19, 20]

^6^The number of past year discriminatory experiences was calculated based on the Everyday Discrimination Scale, where the sum total responses were weighted for each participant to capture the annual chronicity of discrimination experiences [18].

**Fig. 1:**
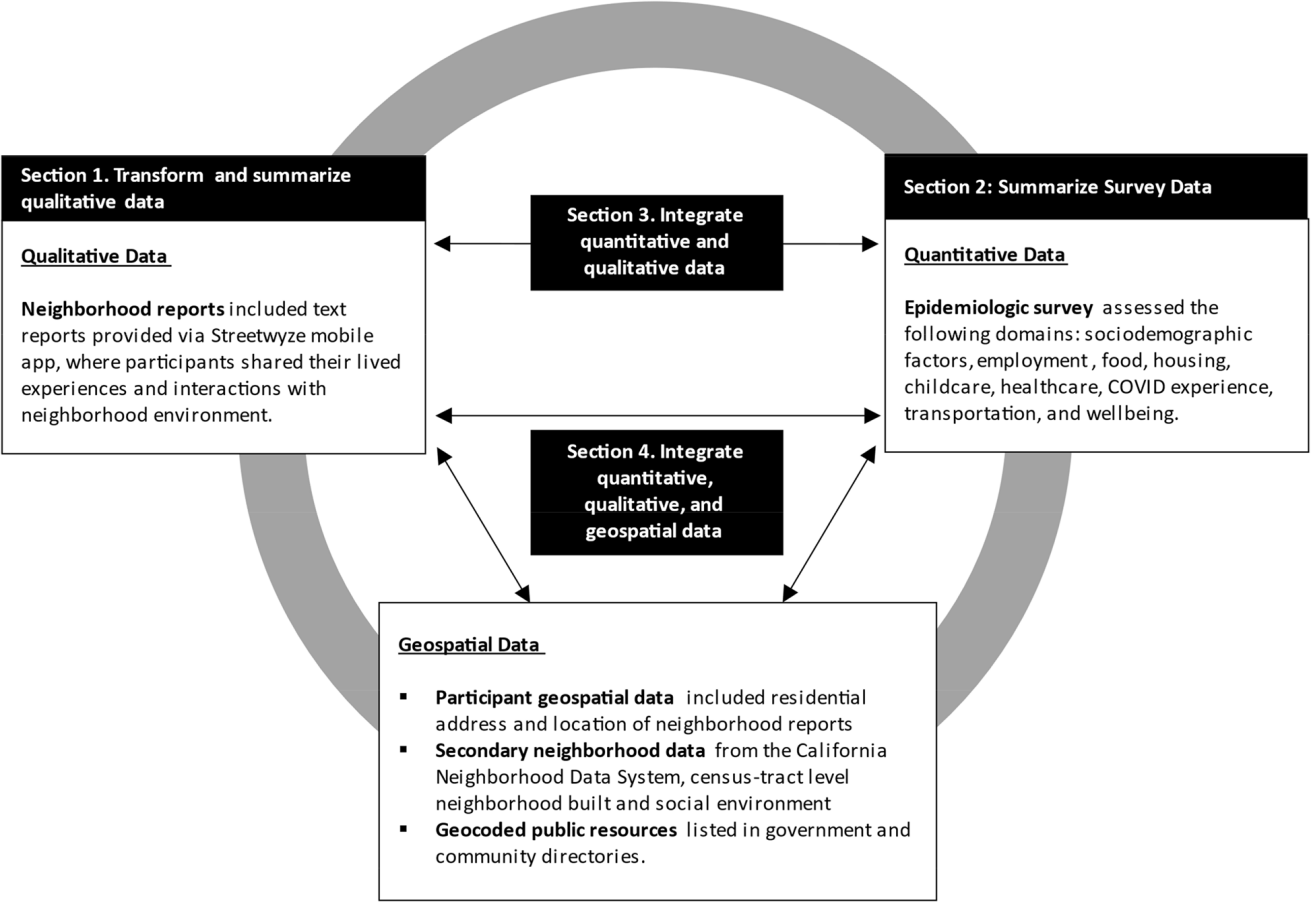
Data types and descriptions, data analysis, and data integration

Our data integration approach is illustrated in Fig. 1 (Subsections below correspond to number boxes in Fig. 1).

### Food experiences in neighborhoods: Summary of transformed qualitative data

Of the 236 qualitative reports collected from 19 participants, the most frequently mentioned parent themes were *access* (61%) and *infection control* (59%) while the least common were *infrastructure* (11%) and *well-being* (5%). The nine parent themes identified through qualitative analysis and their definitions are summarized in Table 2. Frequencies of sub-themes within each of the parent themes is presented in Supplemental Fig. 2. *Food* was the most frequently mentioned sub-theme within the parent themes of *resources* (49%) and *goods* (68%).

**Table 2:** Summary of parent themes

Parent Theme	Definition
Food Access & Availability	Experiences and perceptions related to the physical and economic ability to obtain adequate, culturally appropriate, and nutritious food
Employment & Income Changes	Reports of job loss, reduced hours, wage changes, or other employment disruptions that impacted household income
Housing Stability	Experiences with housing insecurity, eviction risk, or difficulty maintaining stable housing during the pandemic
Healthcare Access	Barriers or facilitators to obtaining medical care, medications, or preventive services
Everyday Discrimination	Incidents or perceptions of unfair treatment based on race, ethnicity, gender, socioeconomic status, or other personal characteristics
Pandemic-Related Stressors	Broader COVID-19 impacts, including childcare disruption, illness, caregiving demands, or mental health strain
Neighborhood Resources	Availability and use of local programs, services, or community supports (e.g., food banks, public health services, outdoor spaces)
Equity & Inclusion	Perceptions of fairness, inclusion, and representation in community programs and decision-making
Coping & Adaptation	Strategies used by individuals or communities to manage challenges and stressors

Parent themes and definitions derived from qualitative analysis of participant neighborhood reports and survey responses, capturing key domains of social determinants of health and lived experiences during the COVID-19 pandemic

**Fig. 2:**
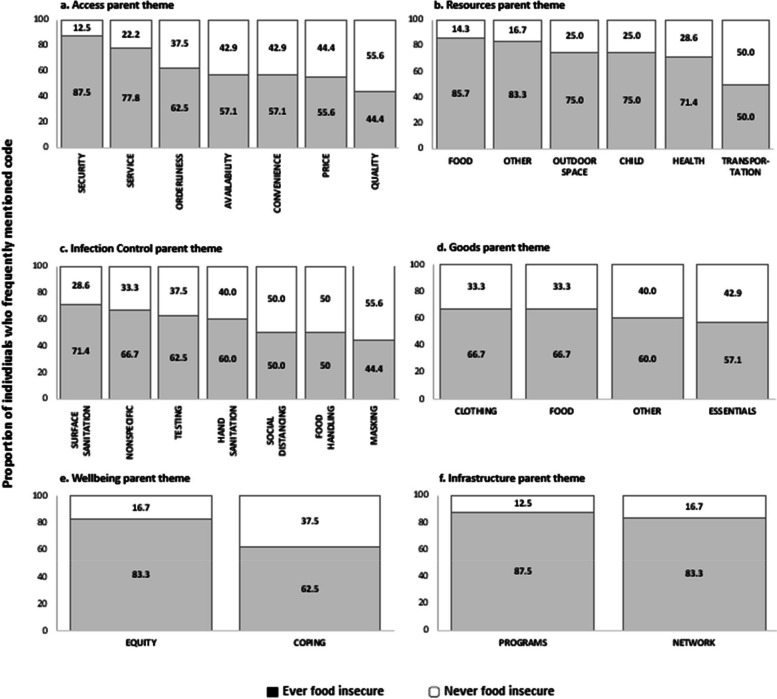
Distribution of frequently mentioned codes within each parent theme based on whether individuals reported food insecurity via survey responses

### Participants circumstances around food access: Summary of survey data

The majority of participants (78.4%) reported food insecurity during either the pre-pandemic or pandemic period (food insecure ever) and 66.7% reported food insecurity in both periods (food insecure persistent) (Table 1). In the period after the pandemic began, 65% of participants reported receiving reduced price or free food resources. The most common resources used were from non-profit organizations (43%, including Meals on Wheels and food banks), government food assistance programs (22%, including CalFresh, the Special Supplemental Nutrition Program for Women, Infants, and Children, or the pandemic Electronic Benefit Transfer (P-EBT) program), and free meals through schools (31%).

### Intersections of individual experiences and neighborhood food experiences: Integrating neighborhood reports and survey responses

An illustrative integration of qualitative and quantitative data is displayed in Fig. 2. For each of the parent themes, the distributions of frequently mentioned codes in qualitative reports are presented according to self-reported food insecurity (ever vs. never food insecure). Most mentions of social and resource programs (87.5%) and informal networks (83.3%) were among participants who were food insecure (12.5% of mentions of resource programs and 16.6% of mentions of informal networks were among those who were not food insecure). Mentions of programs and networks were not specific to food; they include mentions of healthcare, childcare, and community-based organizations (e.g., organizations providing legal assistance). Within the Access parent category, mentions of sub-themes among those who were food insecure ranged from 87.5% of the total mentions of quality of goods to less than 45% of the total mentions of security or safety (compared to 12.5% of the total mentions of quality of goods and 55% of the total mentions of security among those who were not food insecure). These differences may indicate the priority of concerns among participants who are food insecure.

**Fig. 3:**
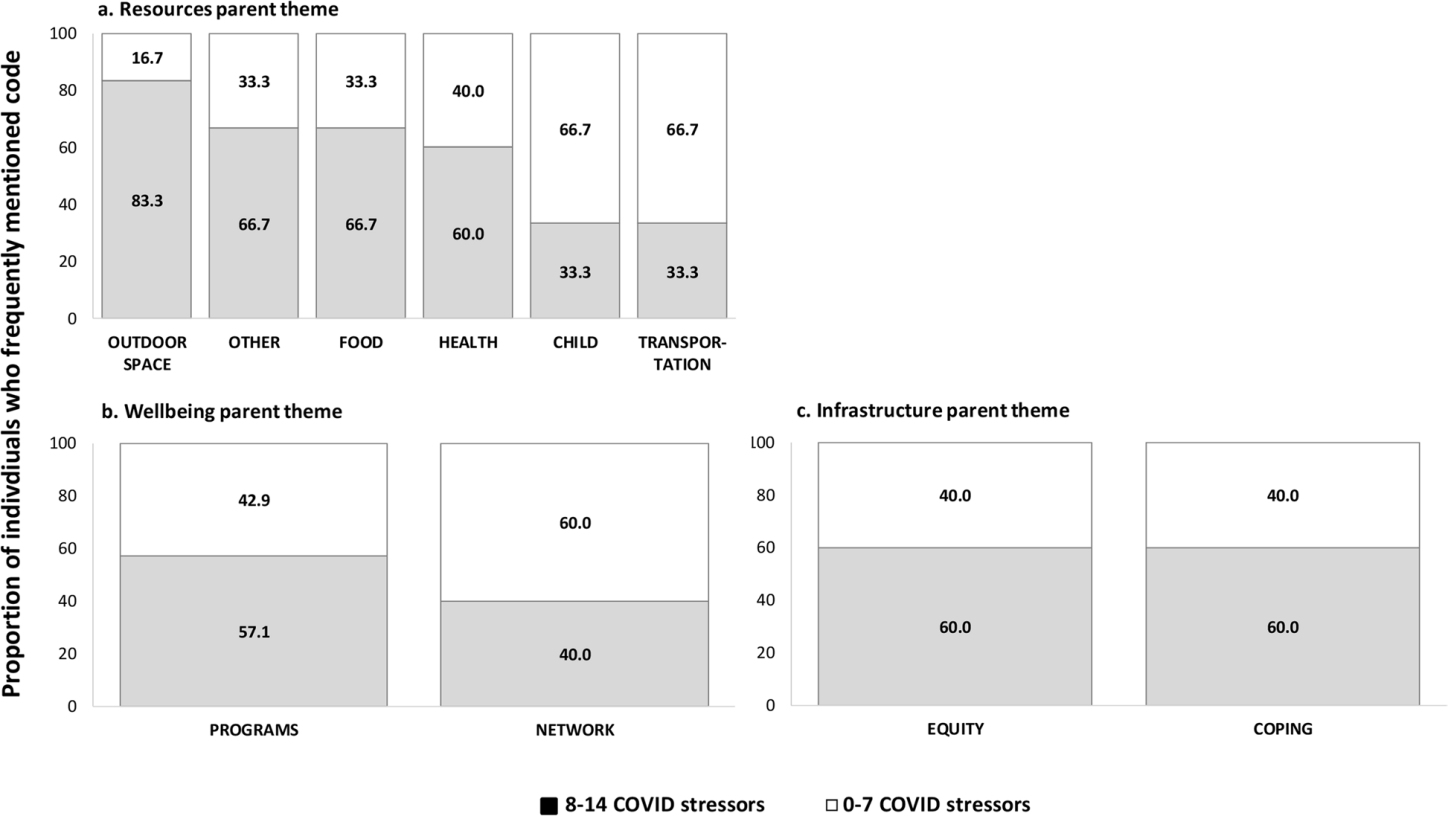
Distribution of frequently mentioned codes within (**a**) resources, **b** infrastructure and (**c**) well-being by reported number of COVID-19 stressors, among those who ever reported food insecurity

Figure 3 presents the frequency of themes mentioned *among* participants who were ever food insecure according to the number of pandemic-related stressors reported, categorized as below (< 8) and above (>/= 8) the average number of stressors among the total participant population. Those who reported food insecurity and >/= 8 pandemic-related stressors had a higher proportion of reports mentioning outdoor space (83.3%), food (66.7%), and health (60.0%) resources; programs and solutions (57.1%); and experiences related to equity (60.0%) and coping (60.0%) compared to those who reported fewer than 8 stressors (Fig. 3). Participants who were food insecure and had >/= 8 pandemic-related stressors, on the other hand, were less likely to mention child or transport resources.

### Locating food experiences: Integrating neighborhood reports, survey responses, and geospatial measures

**Fig. 4:**
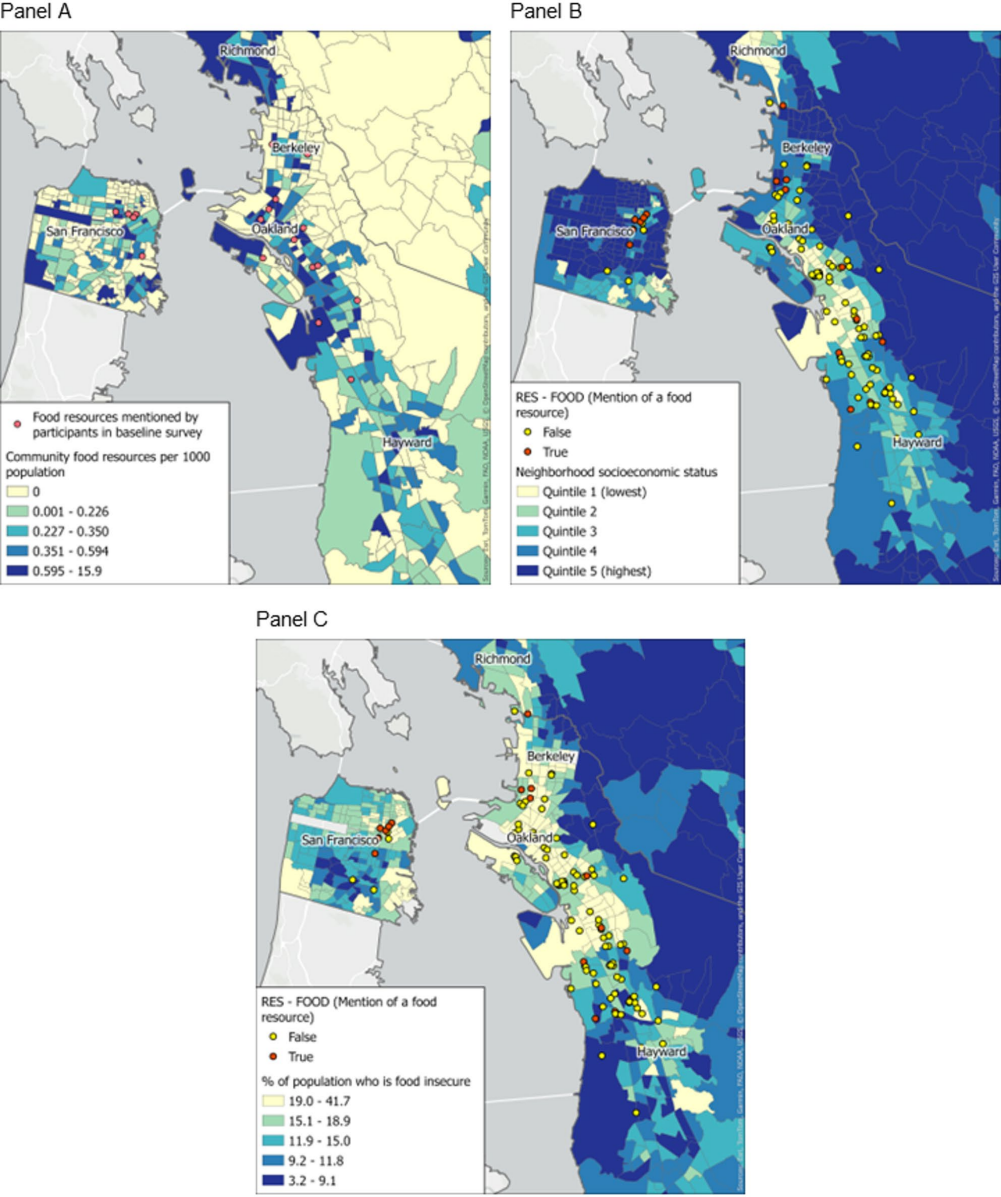
Geospatial integration of participant qualitative and quantitative data

Figure 4 presents strategies for integrating geospatial data with other data types within the domain of food security and food access. In Fig. 4a, neighborhood food resources reported by participants via survey are located over the density of food resources as reported by public resource directories. The food resources that participants reported utilizing appeared to be located within census tracts with a higher density of food resources. In Fig. 4b and 4c the location of qualitative reports mentioning food resources are overlayed on tract-level neighborhood socioeconomic status (4b) and the proportion reporting food insecurity (4c). In general, it appears that neighborhood reports mentioning food resources occurred in census tracts with lower nSES and higher proportions of food insecurity.

## Discussion

Our findings that food insecurity remained highly prevalent before and after the COVID-19 pandemic, with persistent and disproportionate impact on Black, Hispanic, and immigrant populations, align with prior studies documenting similar inequities [4, 44]. Moreover, our findings of high food insecurity among Asian Americans compared to White counterparts suggest similar disproportionate impact on this group. Previous studies do not support this particular finding, but our and other studies similarly lack the ability to disaggregate the Asian American racial/ethnic category according to ethnicity due to small sample sizes, so definitive conclusions cannot reliably be made [4, 44].

Consistent with previous research, we observed that food insecurity clustered with other social stressors and discriminatory experiences, supporting conceptual models linking structural racism, socioeconomic disadvantage, and under-resourced neighborhood food environments to disparities in essential resource access [45–47]. Our geospatial analyses mirror earlier work showing that neighborhood-level “hot spots” of food insecurity coincide with areas of high social vulnerability, where barriers such as limited transportation, inconvenient food pantry hours, and language obstacles are common [5, 48].

By integrating qualitative neighborhood reports, quantitative survey responses, and geospatial mapping, this MMCBPR pilot study demonstrates how multi-modal data can identify targeted needs and resources emerging from community experiences. Participants who had ever experienced food insecurity (before or after the pandemic began) were more likely to report employment, housing, and healthcare changes, as well as everyday discrimination, and more likely to mention resources, programs, and equity-related neighborhood experiences. These findings underscore how place-level disadvantage amplifies food insecurity beyond individual risk factors and emphasize the role of neighborhood context in shaping both actual and perceived inequities in resource distribution.

We observed a higher number of discriminatory experiences among those who experienced food insecurity compared with the overall sample, suggesting a potential link between systemic discrimination and difficulty accessing food for racially and ethnically minoritized groups. Structural racism influences employment, housing stability, healthcare access, and neighborhood food environments, factors that can intensify the effects of crises such as COVID-19 on food security and create layered barriers to meeting basic needs. These results echo prior work showing that discrimination-related stress can exacerbate health inequities and hinder access to community resources [49–51].

Interestingly, some of the highest-SES quintile neighborhoods in our sample (seen in Figs. 4b and 4c) also had high rates of food insecurity. Like findings from disaster and economic disruption research [4, 52]; this pattern suggests that gaps in emergency response and public resource distribution may leave needs unmet for all communities, regardless of socioeconomic resources. Localized supply chain disruptions, sudden changes in household income, and limited eligibility for assistance programs could contribute to this phenomenon.

In addition, participants who mentioned food in neighborhood reports and who had many COVID-related stressors suggested areas for resource development related to outdoor space and health. Thus, a multilevel response to this need might include 1-community food service organizations serving as a point of contact for individuals who may benefit from other resources (healthcare and public health information) and 2-investment in outdoor spaces as sites to promote physical and mental health as well as deliver tangible health resources. Similarly, this observation stresses the importance of cross-institutional communication and connectivity to alleviate the administrative burden experienced by those who access multiple social services.

While this report is focused on food insecurity, it is intended to serve as an illustrative example of how this data model can inform tangible solutions across domains of structural and social determinants of health. For example, we found that those reporting both food insecurity and a higher number of other pandemic-related stressors had higher mentions of resources, programs, potential solutions, and issues related to equity and coping. This suggests that more comprehensive wrap-around services are needed for individuals who already experience stressors related to food insecurity.

In our study, results from maps largely serve to validate the congruence of information across our data sources (e.g., mentions of food occurring most frequently in neighborhoods with higher area-level food insecurity). While we are reassured in the capacity for transformation of neighborhood reports to align with other measures of food access or experience, integration of reports or survey data with geospatial data would be most powerful when it contributes to new or emerging understanding of communities’ needs. Doing so may require smaller geographic units of analysis with larger numbers of community residents contributing reports and survey responses. For example, emergence of themes related to (non-COVID) safety in the vicinity of an existing food resource may be an early signal of an emerging access barrier or relatively frequent positive mentions of racial equity may identify exemplary aspects of existing programs. And of course, new mentions of needed resources may indicate specific areas with emerging need. We will continue to develop the relevancy of our data model to hyper-local assessment of barriers and solutions in ongoing and future studies.

A limitation inherent to our data model is that integration across data sources inevitably reduces cell sizes. Specifically, because participants were provided with multiple options to share their experiences and perspectives, there was limited overlap between data sources. This could lead to an overreliance on making inferences based on single data sources rather than on integration. Despite that, using a data integration model can help overcome the limitations of small sample sizes by leveraging information across sources of data to reinforce the validity of each. For instance, the fact that there were more mentions of food resources in qualitative neighborhood reports among those reporting food insecurity in the epidemiologic survey bolsters the validity of both data sources. While there was some overlap between the 51 quantitative survey respondents and the 19 qualitative participants, allowing for within-participant corroboration, findings should be interpreted cautiously, and further research with a larger population is needed to confirm and expand these results.

Our secondary measures of nSES and food insecurity preceded our other geospatial data and participant address data, which in certain contexts would represent a study limitation. In our case, the 2017 nSES and food insecurity measures were the most recent available at the time we conducted our study, in part due to data delays during the pandemic. While census tract boundaries may have shifted slightly between 2010 and 2010 and while there may have been some shift in census tracts across nSES or food insecurity quintile, the areas (adjacent census tracts) of San Francisco and the East Bay that had relatively low nSES or high food insecurity in 2017 and 2021 were stable [37]. Nevertheless, studies leveraging this data model should also be mindful of the timing of collection across data types.

Due to the small size of the participant population in this pilot study, we were unable to examine results according to individual-level factors such as race, ethnicity, immigration status, LGBTQA + status, and other individual social factors that impact individuals’ vulnerability to structural oppression. Targeted data collection to specific populations where appropriate or increasing participation would allow us to better represent those with intersecting identities. Also due to the small sample size, thematic saturation may not have been reached for some topics. We note that this was a pilot MMCBPR study designed to assess the feasibility of integrating survey, qualitative, and geospatial data rather than to achieve statistical power for hypothesis testing. The sample size was determined by the number of eligible Streetwyze users in the study area during the recruitment period and the project’s capacity to manage and analyze multi-modal data. Pandemic-related barriers, including caregiving and work demands, illness, and technology challenges, combined with the time-intensive nature of community-based research contributed to 32% attrition despite only one formal data collection point.

Despite these limitations, there are several key benefits of this approach to data collection compared with other approaches that have been employed. First, collecting qualitative data using a mobile platform combined with brief, focused survey questions, simultaneously with geographic information, allows for continued participation compared with longer forms of data collection (e.g., in-depth interviews). Additionally, because the Streetwyze platform allows participants to share their feedback and experiences in real-time and within the context of their own neighborhood experiences, the barrier to participation is lower. This allows for sustained and low-effort participation and can make research more accessible for more diverse people and experiences.

## Conclusions

Overall, we have demonstrated the feasibility of simultaneous, multi-modal data collection, which can be scaled to allow for a wider scope of inquiry, along with a tangible model for data integration with the potential to identify salient needs emerging from communities. The combined analysis of quantitative, qualitative, and geospatial data can be adapted by community organizations, policy makers, and researchers to improve the implementation of policies and services over time by periodically tracking and analyzing residential reports and sustaining community participation. This effort is in line with our broader intent to facilitate community-led research and community ownership of data that generates local knowledge, investment, and progress (Akom, Hope Hassberg, and Cruz; pending book chapter).

## Supplementary Information

Supplementary Material 1.

Supplementary Material 2.

## Data Availability

The datasets generated and/or analyzed during the current study are not publicly available due to community preference but are available from the corresponding authors on reasonable request.

## Abbreviations

ACS: American community survey
BIPOC: Black, indigenous, and people of color
CBPR: Community-based participatory research
EDS: Everyday discrimination scale
GIS: Geographic information system
MMCBPR: Mixed methods, community-based participatory research
nSES: Neighborhood socioeconomic status
P-EBT: Pandemic electronic benefit transfer
REDCap: Research electronic data capture
SDOH: Social determinants of health
SES: Socioeconomic status
USDA: United States department of agriculture
