# Supplementary material for: Inequities in food access during the COVID-19 pandemic: A multilevel, mixed methods pilot study
Source: BMC Public Health. 2026 Jan 14;26:549. doi: 10.1186/s12889-025-25964-3 (PMC12888245; doi:10.1186/s12889-025-25964-3)
Supplement: Supplementary file 1 — Supplementary Material 1. [file 12889_2025_25964_MOESM1_ESM.docx]

Contents

[Introduction 2](#_Toc54009487)

[Employment 3](#_Toc54009488)

[Healthcare 5](#_Toc54009489)

[Housing 7](#_Toc54009490)

[Childcare 9](#_Toc54009491)

[Transportation 10](#_Toc54009492)

[Food 11](#_Toc54009493)

[Well-being 12](#_Toc54009494)

[COVID-19 14](#_Toc54009495)

[Financial/Income 16](#_Toc54009496)

[Residence 16](#_Toc54009497)

1. Hamilton CM, Strader LC, Pratt JG, et al. The PhenX Toolkit: get the most from your measures. *Am J Epidemiol*. 2011;174(3):253-260. doi:10.1093/aje/kwr193
2. PROMIS Global Physical Health: 2010-2016 PROMIS Health Organization and PROMIS Cooperative Group https://www.healthmeasures.net/index.php. PROMIS Scale v1.2 - Global Physical 2a 07September2016.
3. PROMIS Global Mental Health: 2010-2016 PROMIS Health Organization and PROMIS Cooperative Group https://www.healthmeasures.net/index.php. PROMIS Scale v1.2 - Global Mental 2a 07September2016.
4. EPII: Grasso DJ, Briggs-Gowan MJ, Ford JD, Carter AS. *The Epidemic - Pandemic Impacts Inventory (EPII).* University of Conneticut School of Medicine; 2020.
5. Everyday Discrimination: Williams DR, Yan Yu null, Jackson JS, Anderson NB. Racial Differences in Physical and Mental Health: Socio-economic Status, Stress and Discrimination. *J Health Psychol*. 1997;2(3):335-351. doi:10.1177/135910539700200305
6. Medical Mistrust: LaVeist TA, Isaac LA, Williams KP. Mistrust of Health Care Organizations Is Associated with Underutilization of Health Services. *Health Serv Res*. 2009;44(6):2093-2105. doi:10.1111/j.1475-6773.2009.01017.x
7. COVEX: Fisher PW, Desai P, Klotz J, et al. COVID-19 Experiences (COVEX). Published 2020. https://www.phenxtoolkit.org/toolkit_content/PDF/Fisher_COVEX.pdf
8. Household Pulse: US Census Bureau. Measuring Household Experiences during the Coronavirus Pandemic. Census.gov. Accessed July 17, 2023. https://www.census.gov/householdpulsedata
9. CRISIS: The CoRonavIruS Health Impact Survey (CRISIS). The PhenX Toolkit. Accessed July 17, 2023. https://www.phenxtoolkit.org/toolkit_content/PDF/CRISIS_Baseline_Adult.pdf
10. COPE: COPE Survey – All of Us Research Hub. Accessed July 17, 2023. https://www.researchallofus.org/data-tools/survey-explorer/cope-survey/
11. CEFIS: Kazak AE, Alderfer M, Enlow PT, et al. COVID-19 Exposure and Family Impact Scales: Factor Structure and Initial Psychometrics. *J Pediatr Psychol*. 2021;46(5):504-513. doi:10.1093/jpepsy/jsab026
12. CAIR: Complementary and Integrative Research. CAIR Pandemic Impact Questionnaire-C-PIQ. https://www.phenxtoolkit.org/toolkit_content/PDF/CAIR_PIQ.pdf
13. CIHWS: University of Texas, Rio Grande Valley. COVID-19 Impact on Health and Wellbeing Survey (CIHWS). https://www.phenxtoolkit.org/toolkit_content/PDF/UTRGV_CIHWS.pdf
14. JHU: Johns Hopkins University. JHU COVID-19 Community Response Survey. https://www.phenxtoolkit.org/toolkit_content/PDF/JHU_C4WARD.pdf
15. MACS-WIHS: MACS-WIHS Combined Cohort Study. MACS-WIHS Questionnaire Baseline COVID-19 Abbreviated Questionnaire BLCOVID. Published April 6, 2020. https://www.phenxtoolkit.org/toolkit_content/PDF/MACS-WIHS.pdf

## Introduction

Thank you for participating in this survey. Your responses will help us to understand the impact of the COVID-19 pandemic on you. Your responses will be used to help improve the resources you need to care for yourself and your family. You will be asked questions on topics such as employment, food access, housing, healthcare, and wellbeing. You can skip any questions you do not feel comfortable answering.

## Informed Consent

Do you have a participant ID number (number starting with 8------)?

- Yes
- No

If yes, what is your participant ID? _______________

- Skip to “Employment section” after providing participant ID

If no, insert informed consent and questions

## Demographic questions

[skip this section if participant ID is provided]

Do you reside in one of the following counties?

- Alameda
- Contra Costa
- San Francisco
- I do not reside in one of these counties 🡪 NOT eligible

What is your age range?

- Below 18 🡪 NOT eligible
- 18 – 24
- 25 – 34
- 35 – 44
- 45 – 54
- 55– 64
- 65 or older

Which of the following best describes your race or ethnic background? (Mark all that apply)

- White
- Hispanic or Latino
- Black or African American
- Asian
- Middle Eastern or North African
- Native Hawaiian or other Pacific Islander
- American Indian or Alaska Native
- Something else, please specify: _________________

How would you describe yourself? (Mark one answer)

- Male
- Female
- Trans male/trans man
- Trans female/trans woman
- Genderqueer/gender non-conforming
- Different identity (please state): _________________

What is your email address?

Your email address will be used to send you a digital gift card upon completion of this survey.

## Employment

**The questions in this section will be related to your employment situation before the pandemic began and now. First, please answer the following questions regarding your employment situation in February 2020 (before the COVID-19 pandemic began).**

1. In February 2020, were you employed?

- Yes **🡪 ask question 1b**
- No **🡪 ask question 1a**

1a. What was the reason you were not employed?

- I am unable to work for health reasons.
- I was caring for someone else/others, including children or adult family members.
- I was looking for a job, but did not have one at the time.
- I am retired.
- Something else, please specify: _______________________

1b. Considering all jobs you had in February 2020, how many paid hours did you typically work per week:

- Less than 20 hours (‘part time’)
- 20-39 hours
- 40 hours (or ‘full-time’)
- More than 40 hours

1c. What setting(s) were you working in February 2020 (before the COVID-19 pandemic began)? (Mark all that apply.) (COVEX)

- At home
- In a medical setting (hospital, clinic, doctor’s office, urgent care center, etc.)
- In an office
- In a private household(s) (nanny, housekeeper, etc.)
- In a setting with regular customer interaction (delivery, transport, retail, food service, restaurant, etc.)
- In the community as a first responder (police, EMS, firefighter, etc.)
- In a warehouse or factory
- In a classroom setting
- Outside (gardening, construction, road work, etc.)
- Something else, please specify: __________________________________

2. In February 2020, were you enrolled in school as a student?

- Yes
- No

**In the next set of question, please consider your employment situation since the pandemic began in March 2020.**

3. Since the pandemic began in March 2020… (Mark all that apply.)

- I moved to working remotely or from home.
- I had a hard time making the transition to working from home.
- I had a hard time doing my job well because of needing to take care of people in my home.
- I reduced my work hours.
- I increased my work hours.
- My workload or work responsibilities increased.
- I lost my job permanently.
- I left my job voluntarily.
- I lost my job temporarily or was not told for how long.
- My job put me at risk of getting COVID.
- I got a new job.
- There were no changes. **🡪 skip to question 5**

4. What setting(s) are you currently working in? (Mark all that apply.) (COVEX)

- **My work settings have not changed since the pandemic began in March 2020**
- At home
- In a medical setting (hospital, clinic, doctor’s office, urgent care center, etc.)
- In an office or apartment building
- In a private household(s) (nanny, housekeeper, etc.)
- In a setting with regular customer interaction (delivery, transport, retail, food service, restaurant, etc.)
- In the community as a first responder (police, EMS, firefighter, etc.)
- In a warehouse or factory
- In a classroom setting
- Outside (gardening, construction, road work, etc.)
- Something else, please specify __________________________________

5. Considering all jobs you currently have, how many paid hours do you typically work per week

- Less than 20 hours (‘part time’)
- 20-39 hours
- 40 hours (or ‘full-time’)
- More than 40 hours

6. Have you reported in person to your work site in the last week? (CIHWS)

- Yes
- No

7. Since March 2020, have you received any of the following sources of income support? (Please mark all that apply.)

- Unemployment insurance payment
- Paid time off from your employer (may be administrative leave if your employer was closed or short-term disability leave)
- Something else, please specify: ___________________________________________________
- I began receiving government assistance as a form of income continuation (example: disability, SNAP or SSI)
- I have not received income support

8. Are you currently enrolled in school as a student?

- Yes
- No

**The next set of questions asks you to consider other individuals in your household.**

9. Is there anyone else (besides yourself) in your household who, in February 2020 contributed to household income? **YES/NO**

- Yes
- No **🡪 skip to question 10**

9a. Thinking of the other individuals in your household who contributed to your household income in February 2020, please indicate whether each of the statements below were true for at least one individual other than yourself. (Mark all that apply.)

- They moved to working remotely or from home.
- They reduced their work hours.
- They increased their work hours.
- They lost their job permanently.
- They left their job voluntarily.
- They lost their job temporarily or was not told for how long.
- Their job put them at risk of getting COVID-19.
- They got a new job.
- There were no changes.

9b. Since March 2020, has anyone in your household received any of the following sources of income support? (Please mark all that apply.)

- Unemployment insurance payment.
- Paid time off from their employer (may be administrative leave if your employer was closed or short-term disability leave)
- They began receiving government assistance as a form of income continuation (example: disability, SNAP or SSI)
- They have not received income support
- Something else, please specify: __________________________________________________

## Healthcare

**The questions in this section are related to your access and use of healthcare.**

10. In February 2020 (before the COVID-19 pandemic began), where did you usually go for routine medical care (seeing a doctor for any reason)? [Select one]

- Community health center or clinic (including free clinic)
- Urgent care clinic /Hospital (not emergency room)
- Private doctor’s office/Kaiser/HMO/PPO
- Emergency room
- Veteran’s Affairs/VA/Military Facility
- Other type of location (please specify other type) ______________________________________
- No usual source of care

11. Has where you usually receive care changed since the pandemic began in March 2020?

- Yes
- No **🡪 skip to question 12**

11a. Where do you currently go for routine medical care (seeing a doctor for any reason)? [Select one]

- - Community health center or Community clinic (including free clinic)
  - Urgent care clinic /Hospital (not emergency room)
  - Private doctor’s office/Kaiser/HMO/PPO
  - Emergency room
  - Veteran’s Affairs/VA/Military Facility
  - Other type of location (please specify other type) ______________________________________
  - No usual source of care

12. In February 2020 (before the COVID-19 pandemic began), what kind of health insurance or health care coverage did you have? [Mark all that apply]

- Insurance provided through my current or former employer or union (including HMO, such as Kaiser, Health Net, Anthem, etc.)
- Insurance provided by another family member (e.g., spouse) through their current or former employer or union (including HMO, such as Kaiser, Health Net, Anthem, etc.)
- Insurance purchased directly from an insurance company (by you or another family member)
- Insurance purchased from an exchange (sometimes called Obamacare or the Affordable Care Act)
- Medi-Cal or other state provided insurance
- Medicare/government insurance
- VA (including those who have ever used or enrolled for VA health care)
- Indian Health Service
- I did not have any medical insurance
- Something else. Please specify: ____________________________________________________________

13. Has your health insurance changed since the pandemic began in March 2020?

- Yes
- No **🡪 skip to question 14**

13a. What kind of health insurance or health care coverage do you currently have? (Mark all that apply)

- - Insurance provided through my current or former employer or union (including HMO, such as Kaiser, Health Net, Anthem, etc.)
  - Insurance provided by another family member (e.g., spouse) through their current or former employer or union (including HMO, such as Kaiser, Health Net, Anthem, etc.)
  - Insurance purchased directly from an insurance company (by you or another family member)
  - Insurance purchased from an exchange (sometimes called Obamacare or the Affordable Care Act)
  - Medi-Cal or other state provided insurance **🡪 if marked, ask question 13a.i.**
  - Medicare/government insurance
  - VA (including those who have ever used or enrolled for VA health care)
  - Indian Health Service
  - I do not have any medical insurance
  - I don’t know
  - Something else. Please specify: ____________________________________________________________

13.a.i. Did you gain insurance as a part of emergency coverage or Medicaid expansion?

- - - Yes
    - No
    - Not sure

**The next set of questions asks about the ways in the COVID-19 pandemic may have affected your access to medical care since March 2020.** (adapted from WIHS-MACS)

14. Have missed needed or planned medical appointments?

- Yes
- No **🡪 skip to question 15**

14a. Why were you unable to attend these appointments? (Mark all that apply.)

- The healthcare facility was closed because of the COVID-19 pandemic.
- You had no transportation to get to the healthcare provider's office.
- You couldn’t afford payment for the healthcare appointment.
- Your appointment was converted to a virtual visit, and you couldn’t attend. **🡪 if marked, ask question 14a.i.**
- You were concerned about your COVID safety during an in-person visit.
- Something else, specify: __________________

14a.i. You indicated in the previous question that you were unable to attend a virtual health visit. Why were you not able to attend? ________________________________________________________________________________________

14b. Were any of these missed appointments for prenatal care for yourself?

- - Yes
  - No

15. Have you been unable to obtain medications that you normally take or need?

- Yes
- No **🡪 skip to question 16**

15a. Why were you unable to obtain medications? (Mark all that apply.) Was it because…?

- The healthcare facility was closed because of the COVID-19 pandemic.
- You had no transportation to get obtain your medication.
- You couldn’t afford the medication
- The medication was no longer available
- You were concerned about your COVID safety during an in-person visit.
- Something else, specify: __________________

## Housing

**The following questions are about your current living situation and the individuals who live with you.**

16. Where do you live? Select only one answer. (JHU)

- House/condo/townhouse **🡪 if marked, ask 16a.**
- Apartment
- Dormitory
- Assisted living facility
- Skilled nursing center
- No consistent primary residence **🡪 if marked, ask 16b.**
- Something else. Please specify: __________ **🡪 if marked, ask 16b.**

16a. Is your house or apartment…? Select only one answer. (CENSUS)

- Owned free and clear?
- Owned with a mortgage or loan (including home equity loans)?
- Rented?
- Occupied without payment of rent?

16b. What is your usual nighttime accommodation? Select only one answer. (JHU)

- Shelter
- Transitional housing/safe haven
- Street/outside/tent/encampment
- Abandoned building/squat
- Vehicle (car, van, RV, camper)
- Hotel or motel
- Something else. Please specify __
- Prefer not to say

17. Including yourself, how many individuals have been living in your household most of the time since March 2020 (since the COVID-19 pandemic began)? By household, we mean individuals (adults and children) who live together in the same dwelling. (adapted from COVEX)

____________ People (write “1” if living alone)

18. Has there been a change in where you live or who you live with since the pandemic began in March 2020? This could mean temporarily living in a different place or a change in the people you live with. (adapted from COVEX)

- Yes
- No **🡪 skip to question 19**

18a. How has your living situation changed? (Mark all that apply.) (adapted from COVEX)

- Moved in with “parents” or into family home (e.g., returned home from school, etc.).
- Moved to a different location/situation (e.g., moved in with other family members or friends).
- Other people added to household.
- People moved out of your household.
- Moved away from your family.
- Lost housing.
- Moved into a shelter.
- Other _____________________________________

19. Since the COVID-19 disease pandemic began, have you/your family been unable to pay important bills like rent or utilities? [modified from EPII]

- Yes
- No

20. Have you received financial assistance meant for your rent or mortgage payment?

- Yes
- No **🡪 skip to question 21**

20a. Please specify from whom you have received this assistance: ___________________________________________________

21. Considering the past two weeks, please mark if each of the following statements were true for you. (Mark all that apply)

- If I were exposed to COVID-19, I have the resources available to stay in my home for at least 15 days. (adapted from CIHWS)
- I only leave my home for essential reasons (job, food, medications, and other home supplies. (adapted from CIHWS)

22. During the past two weeks, to what degree were you concerned about the stability of your living situation? (EPII)

- Not at all
- Slightly
- Moderately
- Very
- Extremely

23. Are you familiar with the ‘eviction moratorium’ in California (this is a temporary program preventing tenants from being evicted due to an inability to pay their rent or mortgage)? (created)

- Yes
- No **🡪 skip to question 24**

23a. Have you used the eviction moratorium to avoid eviction or foreclosure? This may mean submitting a declaration of COVID hardship in response to ‘pay or quit’ or vacate notice from your landlord or requesting a forbearance to avoid a foreclosure timeline)? (created)

- - Yes
  - No

## Childcare

**You are about halfway through the survey. We would like to ask a few questions about how your experiences as a caretaker or household with children have been shaped by the COVID-19 pandemic.**

24. Are you responsible for the care (for example, daily caretaking, financial care, or supervision) of children in your household?

- Yes
- No **🡪 skip to next section**

25. At the beginning of the COVID-19 pandemic (March 1, 2020), how many children in your household were between the ages of:

0-4 years: ____________

5-9 years: ____________

10-14 years: ____________

15-17 years: ____________

26. Did you utilize either free/or paid childcare prior to the COVID-19 pandemic?

- Yes
- No **🡪 skip to next section**

26a. Since March 2020, please indicate whether each of the statements below have been true for you. (Mark all that apply.)

- Our schools/childcare centers closed. (CEFIS)
- I had difficulty arranging for childcare. (ECHO)
- I had to utilize a new childcare option. (created)
- I had to pay more for childcare. (ECHO)
- I or another person in the household had to change our work schedule or multi-task in order to care for our children ourselves. (ECHO)
- My regular childcare has not been affected by the COVID-19 outbreak. (ECHO)

## Transportation

**In the following section, we will ask questions related to transportation.**

27. What was your primary mode of transportation in February 2020 (before the COVID-19 pandemic began)?

- Personal vehicle
- Carpool
- Public transportation **🡪 if marked, ask 27a**
- Car rideshare services
- Bicycle (your own or shared services)
- Walking

27a. What transportation services did you use? (Mark all that apply)

- Bay Area Rapid Transit (BART)
- AC transit (buses in the east bay)
- Muni (buses and light rail in San Francisco)
- Contra Costa Transit Agency
- Ferry
- Something else, please specify: _______________________

28. Since March 2020 (since the COVID-19 pandemic began), how has your experience with your primary mode of transportation changed?

- It’s been about the same **🡪 skip to question 29**
- I use it more **🡪 skip to question 29**
- I use it less
- I’ve switched from one mode to another

28a. What caused the change in your primary mode of transportation? (Mark all that apply)

- I have concerns about safety
- My primary mode of transportation is no longer available or is less available now
- Change in my employment situation has affected by transportation needs
- Something else, please specify: ___________________________

## Food

**The questions in this section are related to your access to food.**

29. For the next 3 statements, please indicate whether the statement was often true, sometimes true, or never true for your household in February 2020 (before the COVID-19 pandemic began). (PBRC)

|  | **Often true** | **Sometimes true** | **Never true** | **I don’t know** |
| --- | --- | --- | --- | --- |
| We were worried that the food we had wouldn’t last. |  |  |  |  |
| We couldn’t afford to eat balanced meals. |  |  |  |  |
| We couldn’t get the food that me or my family wanted to eat. |  |  |  |  |

30. For the next 3 statements, please indicate whether the statement is currently true, often true, sometimes true, or never true. (PBRC)

|  | **Currently true** | **Sometimes true** | **Never true** | **I don’t know** |
| --- | --- | --- | --- | --- |
| We are worried that the food we had won’t last. |  |  |  |  |
| We can’t afford to eat balanced meals. |  |  |  |  |
| We can’t get the food that me or my family want to eat. |  |  |  |  |

***If answered “currently true” or “sometimes true” to any of the statements above…***

30a. Why do you sometimes not have enough to eat (or not what you wanted to eat)? [Mark all that apply.] (CENSUS)

- Couldn’t afford to buy more food
- Couldn’t get out to buy food (for example, didn’t have transportation, or had mobility or health problems that prevented me from getting out)
- Afraid to go or didn’t want to go out to buy food
- Couldn’t get groceries or meals delivered to me
- The stores didn’t have the food I wanted

31. Since March 2020, (since the COVID-19 pandemic began), how do you usually purchase groceries?

- In person at a conventional grocery store or supermarket (e.g. Target, Walmart, Safeway, Trader Joes)
- In person at a convenience store (e.g. corner market or liquor store)
- In person at a farmer’s market or other independently run food stand
- Online ordering and delivery (e.g. Instacart, Postmates)
- Some other way. Please specify: ______________________________________________

32. Since March 2020 (since the COVID-19 pandemic began), have you received any reduced or free food resources (from any source, such as the government, community organizations, or individuals)? -

- Yes
- No **🡪 skip to question 33**

32a. Which food resources have you received? (Mark all that apply)

- CalFresh, also known as food stamps

**🡪 if marked, ask**: 32a.i. Have you been able to use your CalFresh as needed? **Y/N**

- WIC (Women, Infants, and Children)

**🡪 if marked, ask**: 32a.ii. Have you been able to use your WIC as needed? **Y/N**

- Emergency food boxes
- Free meals through the school or other programs aimed at children
- Food pantry or food bank

**🡪 if marked, ask**: 32a.iii. Which food pantry or food bank have you received resources from?

- Home-delivered meal service like Meals on Wheels

**🡪 if marked, ask**: 32a.iv. Which home delivered meal service have you received resources from?

- Church, synagogue, temple, mosque or other religious organization
- Shelter or soup kitchen

**🡪 if marked, ask**: 32a.v. Which shelter or soup kitchen have you received resources from?

- Other community program

**🡪 if marked, ask**: 32a.vi. Which other community programs have you received resources from?

- Pandemic EBT (P-EBT)

**--> if marked, ask**: 32a.vii. Have you been able to use your P-EBT as intended?

- Family, friends, or neighbors
- Something else. Please specify: ________________________________________

## Well-being

**In this section, we will ask some questions about your health and well-being.**

33. PROMIS 2-item global physical health, 2-item global mental health

|  |  |  |  |  |  |
| --- | --- | --- | --- | --- | --- |
| In general, how would you rate your physical health? | **Excellent** | **Very good** | **Good** | **Fair** | **Poor** |
| To what extent are you able to carry out your everyday physical activities such as walking, climbing stairs, carrying groceries, or moving a chair? | **Completely** | **Mostly** | **Moderately** | **A little** | **Not at all** |
| In general, how would you rate your mental health, including your mood and your ability to think? | **Excellent** | **Very good** | **Good** | **Fair** | **Poor** |
| In general, how would you rate your satisfaction with your social activities and relationships? | **Excellent** | **Very good** | **Good** | **Fair** | **Poor** |

34. In the last month, how often have you felt: (Perceived stress scale [PSS-4] (WIHS-MACS)

|  | **Never** | **Almost never** | **Sometimes** | **Fairly often** | **Very often** |
| --- | --- | --- | --- | --- | --- |
| That you were unable to control the important things in your life? |  |  |  |  |  |
| Confident about your ability to handle your personal problems? |  |  |  |  |  |
| That things were going your way? |  |  |  |  |  |
| Difficulties were piling up so high that you could not overcome them? |  |  |  |  |  |

35. The following is a list of concerns that some people have had since the pandemic began in March 2020. For you, what, if any, have been great sources of stress or worry for you as a result of the pandemic? (Mark all that apply) (ECHO)

- Concern for my health
- Concern for the health of family members
- Financial concerns
- Impact on work
- Impact on my child
- Impact on my community
- Impact on relationships with adult family members
- Access to food
- Access to baby supplies (e.g., formula, diapers, wipes)
- Access to personal care products or household supplies
- Access to healthcare, including mental health care
- Access to housing (including rent or mortgage responsibility)
- Ability to parent how I want or parenting responsibilities
- Ability to care for older adults or people with disabilities
- Social distancing or being quarantined
- Transportation availability and safety
- Something else. Please specify: ________________
- I am not stressed or worried about the COVID-19 outbreak

36. In your day-to-day life, how often do any of the following things happen to you? (Everyday discrimination scale)

|  | **Almost everyday** | **At least once a week** | **A few times a month** | **A few times a year** | **Less than once a year** | **Never** |
| --- | --- | --- | --- | --- | --- | --- |
| You are treated with less courtesy than other people are |  |  |  |  |  |  |
| You are treated with less respect than other people are |  |  |  |  |  |  |
| You receive poorer service than other people at restaurants or stores |  |  |  |  |  |  |
| People act as if they think you are not smart |  |  |  |  |  |  |
| People act as if they are afraid of you |  |  |  |  |  |  |
| People act as if they think you are dishonest |  |  |  |  |  |  |
| People act as if they’re better than you are |  |  |  |  |  |  |
| You are called names or insulted |  |  |  |  |  |  |
| You are threatened or harassed |  |  |  |  |  |  |

***If answered “A few times a year” or more frequently to at least one question, ask*:**

36a. What do you think is the main reason for these experiences? (Mark all that apply).

- Your ancestry or national origins
- Your gender
- Your race/ethnicity
- Your age
- Your religion
- Your height, weight, or some other aspect of your physical appearance
- Your sexual orientation
- Your education or income level

## COVID-19

**The questions in this section are related to your household’s experiences with COVID-19 illness.**

37. Have you or someone in your household had symptoms of COVID-19 (since March 2020)? Common symptoms of COVID-19 include newly developed fever, dry cough, and general feelings of “tiredness”. Less common symptoms include aches and pains, sore throat, diarrhea, conjunctivitis, headache, loss of taste or smell, or rashes on skin/discoloration of fingers and toes.

- Yes
- No

38. Have you or someone in your household tested positive for coronavirus?

- Yes
- No

39. Have you or someone in your household been hospitalized with COVID?

- Yes
- No

40. Have you or someone in your household been in the ICU (intensive care unit) with COVID?

- Yes
- No

41. Has someone in your household died of COVID?

- Yes
- No

## Financial/Income

**The questions in this section are related to your financial situation.**

42. What was your total household income in 2019? This refers to your income and that of any other adult living in the home.

- Less than $10,000
- $10,000 to $19,999
- $20,000 to $29,999
- $30,000 to $39,999
- $40,000 to $49,999
- $50,000 to $59,999
- $60,000 to $69,999
- $70,000 to $79,999
- $80,000 to $89,999
- $90,000 to $99,999
- $100,000 to $149,999
- $150,000 or more

43. What is your anticipated household income for 2020?

- Less than $10,000
- $10,000 to $19,999
- $20,000 to $29,999
- $30,000 to $39,999
- $40,000 to $49,999
- $50,000 to $59,999
- $60,000 to $69,999
- $70,000 to $79,999
- $80,000 to $89,999
- $90,000 to $99,999
- $100,000 to $149,999
- $150,000 or more

44. The next question is about money you may have saved up in case of an emergency. If you and your household had to live on money you currently have saved and had no other money coming in, how long could you and your household continue living at your current address and continue buying and doing the things you currently do? Using your best guess is fine. Select only one answer.

- Less than a month
- 1 – 2 months
- 3 – 6 months
- 7 – 12 months
- More than 1 year

## Residence

**Address of residence**

This information will be used to inform organizations where resources are needed, or where useful and effective resources are available during the pandemic. Your address will be treated confidentially.

45. What you your usual address of residence?

Street number and street ________________________________

City ____________________________________

Zip code ____________________________________

If you do not want to or are not able to provide your usual address of residence, can you share the nearest cross-streets to your usual address of residence?

Street #1

Street #2

City
