# Supplementary material for: Inequities in food access during the COVID-19 pandemic: A multilevel, mixed methods pilot study
Source: BMC Public Health. 2026 Jan 14;26:549. doi: 10.1186/s12889-025-25964-3 (PMC12888245; doi:10.1186/s12889-025-25964-3)
Supplement: Supplementary file 2 — Supplementary Material 2. [file 12889_2025_25964_MOESM2_ESM.docx]

Contents

[Introduction 2](#_Toc63419325)

[Neighborhood Perceptions (COVID safety, social networks) – 5](#_Toc63419326)

[Employment 11](#_Toc63419327)

[Healthcare 13](#_Toc63419328)

[Housing 15](#_Toc63419329)

[Childcare 17](#_Toc63419330)

[Elder Care 18](#_Toc63419331)

[Transportation 19](#_Toc63419332)

[Food 20](#_Toc63419333)

[Well-being 21](#_Toc63419334)

[Social support 23](#_Toc63419335)

[COVID-19 (NEW VACCINE QUESTIONS, QUSETIONS 39-41) 25](#_Toc63419336)

[Financial/Income 26](#_Toc63419337)

[Residence 27](#_Toc63419338)

1. Neighborhood Safety: Mujahid, M. S., Diez Roux, A. V., Morenoff, J. D., & Raghunathan, T. (2007). Assessing the measurement properties of neighborhood scales: from psychometrics to ecometrics. *American journal of epidemiology*, *165*(8), 858-867.
2. Neighborhood Perceptions: Unpublished sources (personal communication), questions currently in use by ongoing cohort studies.
3. Neighborhood Collective Efficacy, <https://www.phenxtoolkit.org/protocols/view/210801>
4. Elder care: Texas Health and Human Services Commission (HHSC)
5. Social support: The Medical Outcomes Study (MOS) Social Support Survey. Sherbourne, C. D., & Stewart, A. L. (1991). The MOS social support survey. *Social science & medicine*, *32*(6), 705-714.
6. Coping: Coping with COVID-19, COVID-19 Experiences (COVEX). Fisher, P.W., Desai, P., Klotz, J., Turner, J.B., Reyes-Portillo, J.A., Ghisolfi, I., Canino, G., and Duarte, C.S. (2020). COVID-19 Experiences (COVEX). Section 7: Coping. Columbia University: Disaster Lit.
7. Resilience: Brief Resilience Scale (BRS). Smith, B. W., Dalen, J., Wiggins, K., Tooley, E., Christopher, P., & Bernard, J. (2008). The brief resilience scale: Assessing the ability to bounce back. International journal of behavioral medicine, 15(3), 194-200.

Note: Employment, healthcare, housing, childcare, transportation, food, well-being (other than coping and resilience), financial/income, and residence sections are from the baseline survey.

## Introduction

Thank you for participating in this survey. Your responses will help us to understand how the COVID-19 pandemic continues to impact you. Your responses will be used to help improve the resources you need to care for yourself and your family. You will be asked questions on topics such as employment, food access, housing, healthcare, and wellbeing. It will take about 10-15 minutes to complete. You can skip any questions you do not feel comfortable answering.

## Neighborhood Perceptions (COVID safety, social networks)

**Neighborhood perceptions (respond, section c)**

Research increasingly shows that where we live matters in terms of our health. We would like to know a little bit about your experiences in your neighborhood.

C1. How many years have you lived at your current location?

- Less than 1 year
- 1-5 years
- 6-10 years
- 11-15 years
- 16-20 years
- 21+ years

C2. Please mark answers for each of these statements. (Mujahid et al.)

| a. On average, I feel safe walking in my neighborhood, day or night. | - Strongly agree - Agree - Neutral (neither agree nor disagree) - Disagree - Strongly disagree |
| --- | --- |
| b. Violence is not a problem in my neighborhood. | - Strongly agree - Agree - Neutral (neither agree nor disagree) - Disagree - Strongly disagree |
| c. My neighborhood is safe from crime. | - Strongly agree - Agree - Neutral (neither agree nor disagree) - Disagree - Strongly disagree |

C3. As whole, how much of a problem is

|  |  |  |
| --- | --- | --- |
| a. Traffic | | - None/minor problem - Somewhat serious problem - Very serious problem - Don’t know |
| b. A lot of noise | | - None/minor problem - Somewhat serious problem - Very serious problem - Don’t know |
| c. Trash and litter | | - None/minor problem - Somewhat serious problem - Very serious problem - Don’t know |
| d. Too much light at night | | - None/minor problem - Somewhat serious problem - Very serious problem - Don’t know |

C4. Thinking about your neighbors, as a whole:

| a. How often do/did you see neighbors talking outside in the yard, on the street, at the corner park, etc.? | - Often - Sometimes - Rarely/never - Don’t know |
| --- | --- |
| b. How often do/did neighbors watch out for each other, such as calling if they see a problem? | - Often - Sometimes - Rarely/never - Don’t know |
| c. How many neighbors do/did you know by name? | - Often - Sometimes - Rarely/never - Don’t know |
| d. How many neighbors do/did you have a friendly talk with at least once a week? | - Often - Sometimes - Rarely/never - Don’t know |
| e. How many neighbors could you ask for help, such as to “borrow a cup of sugar” or some other small favor? | - Often - Sometimes - Rarely/never - Don’t know |

10. Suppose that because of budget cuts the fire station closest to your home was going to be closed down by the city. How likely is it that neighborhood residents would organize to try to do something to keep the fire station open? (Neighborhood collective efficacy)

- Very likely
- Likely
- Neither likely nor unlikely
- Unlikely
- Very unlikely
- Don’t know
- Refused

**COVID safety** - neighborhood

For this next set of questions, please indicate how concerned you feel about getting sick with COVID-19 while doing the following activities:

|  | Not at all concerned | A little concerned | Somewhat concerned | Very concerned | Extremely concerned | Not applicable |
| --- | --- | --- | --- | --- | --- | --- |
| Using your primary mode of transportation |  |  |  |  |  |  |
| Doing physical activity or exercising outdoors |  |  |  |  |  |  |
| Shopping or running errands |  |  |  |  |  |  |
| Attending my healthcare appointments |  |  |  |  |  |  |
| Obtaining medications |  |  |  |  |  |  |
| Spending time in common/shared spaces around my home (e.g., laundry rooms or common areas) |  |  |  |  |  |  |
| Working at my place of employment |  |  |  |  |  |  |
| Going to childcare settings and schools |  |  |  |  |  |  |

- If moderately/very/extremely concerned are marked, then ask:

Are you concerned about [item above] because…

- People are not social distancing/there are too many people to practice social distancing?
- People in this setting are not wearing masks (or are not wearing them correctly)
- Surfaces in this setting are not properly cleaned or sanitized
- Hand sanitizer/supplies available
- Something else, please specify: ____________________________

## Employment

**The following questions will ask about your current employment status.**

1. Are you currently employed?

- Yes **🡪 ask question 1b**
- No **🡪 ask question 1a**

1a. What is the reason you are not currently employed?

- I am unable to work for health reasons.
- I was caring for someone else/others, including children or adult family members.
- I was looking for a job but did not have one at the time.
- I am retired.
- Something else, please specify: _______________________

1b. How many paid hours do typically work per week?

- Less than 20 hours (‘part time’)
- 20-39 hours
- 40 hours (or ‘full-time’)
- More than 40 hours

1c. In what setting(s) are you currently working? (Mark all that apply.) (COVEX)

- At home
- In a medical setting (hospital, clinic, doctor’s office, urgent care center, etc.)
- In an office
- In a private household(s) (nanny, housekeeper, etc.)
- In a setting with regular customer interaction (delivery, transport, retail, food service, restaurant, etc.)
- In the community as a first responder (police, EMS, firefighter, etc.)
- In a warehouse or factory
- In a classroom setting
- Outside (gardening, construction, road work, etc.)
- Something else, please specify: __________________________________

2. Are you currently enrolled in school as a student?

- Yes
- No

3. Have you reported in person to your work site in the last week? (CIHWS)

- Yes
- No

7. In the last month, have you received any of the following sources of income support? (Please mark all that apply.)

- Unemployment insurance payment
- Paid time off from your employer (may be administrative leave if your employer was closed or short-term disability leave)
- Something else, please specify: ___________________________________________________
- I began receiving government assistance as a form of income continuation (example: disability, SNAP or SSI)
- I have not received income support

**The next set of questions asks you to consider other individuals in your household.**

9. Is there anyone else (besides yourself) in your household who contributes to household income?

- Yes
- No **🡪 skip to question 10**

9b. In the last month, has anyone in your household received any of the following sources of income support? (Please mark all that apply.)

- Unemployment insurance payment
- Paid time off from their employer (may be administrative leave if their employer was closed or short-term disability leave)
- They began receiving government assistance as a form of income continuation (example: disability, SNAP or SSI)
- They have not received income support.

## Healthcare

**The questions in this section are related to your access and use of healthcare.**

10. Currently, where do you usually go for routine medical care (seeing a doctor for any reason)? [Select one]

- Community health center or clinic (including free clinic)
- Urgent care clinic /Hospital (not emergency room)
- Private doctor’s office/Kaiser/HMO/PPO
- Emergency room
- Veteran’s Affairs/VA/Military Facility
- Other type of location (please specify other type) ______________________________________
- No usual source of care

11. What kind of health insurance or health care coverage do you have? [Mark all that apply]

- Insurance provided through my current or former employer or union (including HMO, such as Kaiser, Health Net, Anthem, etc.)
- Insurance provided by another family member (e.g., spouse) through their current or former employer or union (including HMO, such as Kaiser, Health Net, Anthem, etc.)
- Insurance purchased directly from an insurance company (by you or another family member)
- Insurance purchased from an exchange (sometimes called Obamacare or the Affordable Care Act)
- Medi-Cal or other state provided insurance
- Medicare/government insurance
- VA (including those who have ever used or enrolled for VA health care)
- Indian Health Service
- I did not have any medical insurance
- Something else. Please specify: ____________________________________________________________

**The next set of questions asks about the ways in the COVID-19 pandemic may have affected your access to medical care recently.** (adapted from WIHS-MACS)

12. In the past month, have you missed needed or planned medical appointments?

- Yes
- No **🡪 skip to question 13**

12a. Why were you unable to attend these appointments? (Mark all that apply.)

- The healthcare facility was closed because of the COVID-19 pandemic.
- You had no transportation to get to the healthcare provider's office.
- You couldn’t afford payment for the healthcare appointment.
- Your appointment was converted to a virtual visit, and you couldn’t attend. **🡪 if marked, ask question 14a.i.**
- You were concerned about your COVID safety during an in-person visit.
- Something else, specify: __________________

12a.i. You indicated in the previous question that you were unable to attend a virtual health visit. Why were you not able to attend? ________________________________________________________________________________________

12b. Were any of these missed appointments for prenatal care for yourself?

- - Yes
  - No

13. In the past month, have you been unable to obtain medications that you normally take or need?

- Yes
- No **🡪 skip to question 14**

13a. Why were you unable to obtain medications? (Mark all that apply.) Was it because…?

- The healthcare facility was closed because of the COVID-19 pandemic.
- You had no transportation to get obtain your medication.
- You couldn’t afford the medication
- The medication was no longer available
- You were concerned about your COVID safety during an in-person visit.
- Something else, specify: __________________

## Housing

**The following questions are about your current living situation and the individuals who live with you.**

In the past 2 months, have you experienced changes in where you live or who you live with?

- - Yes
  - No 🡪 Skip to 16

14. Where do you live? Select only one answer. (JHU)

- House/condo/townhouse **🡪 if marked, ask 14a.**
- Apartment
- Dormitory
- Assisted living facility
- Skilled nursing center
- No consistent primary residence **🡪 if marked, ask 14b.**
- Something else. Please specify: __________ **🡪 if marked, ask 14b.**

14a. Is your house or apartment…? Select only one answer. (CENSUS)

- Owned free and clear?
- Owned with a mortgage or loan (including home equity loans)?
- Rented?
- Occupied without payment of rent?

14b. What is your usual nighttime accommodation? Select only one answer. (JHU)

- Shelter
- Transitional housing/safe haven
- Street/outside/tent/encampment
- Abandoned building/squat
- Vehicle (car, van, RV, camper)
- Hotel or motel
- Something else. Please specify __
- Prefer not to say

15. Including yourself, how many individuals have been living in your household most of the time in the past month? By household, we mean individuals (adults and children) who live together in the same dwelling. (adapted from COVEX)

____________ People (write “1” if living alone)

16. In the past month, have you/your family been unable to pay important bills like rent or utilities? [modified from EPII]

- Yes
- No

17. In the past month, have you received financial assistance meant for your rent or mortgage payment?

- Yes
- No **🡪 skip to question 21**

17a. Please specify from whom you have received this assistance: ___________________________________________________

18. Considering the past two weeks, please mark if each of the following statements were true for you. (Mark all that apply)

- If I were exposed to COVID-19, I have the resources available to stay in my home for at least 15 days. (adapted from CIHWS)
- I only leave my home for essential reasons (job, food, medications, and other home supplies. (adapted from CIHWS)

19. During the past two weeks, to what degree were you concerned about the stability of your living situation? (EPII)

- Not at all
- Slightly
- Moderately
- Very
- Extremely

20. Are you familiar with the ‘eviction moratorium’ in California (this is a temporary program preventing tenants from being evicted due to an inability to pay their rent or mortgage)? (created)

- Yes
- No **🡪 skip to question 24**

20a. Have you used the eviction moratorium to avoid eviction or foreclosure? This may mean submitting a declaration of COVID hardship in response to ‘pay or quit’ or vacate notice from your landlord or requesting a forbearance to avoid a foreclosure timeline)? (created)

- - Yes
  - No

## Childcare

**You are about halfway through the survey. We would like to ask a few questions about how your current experiences as a caretaker or household with children.**

21. Are you responsible for the care (for example, daily caretaking, financial care, or supervision) of children in your household?

- Yes
- No **🡪 skip to next section**

22. How many children in your household are between the ages of:

0-4 years: ____________

5-9 years: ____________

10-14 years: ____________

15-17 years: ____________

23. In the past month, did you utilize either free/or paid childcare?

- Yes
- No **🡪 skip to next section**

23a. Please indicate whether each of the statements below have been true for you in the past month. (Mark all that apply.)

- Our schools/childcare centers closed. (CEFIS)
- I had difficulty arranging for childcare. (ECHO)
- I had to utilize a new childcare option. (created)
- I had to pay more for childcare. (ECHO)
- I or another person in the household had to change our work schedule or multi-task in order to care for our children ourselves. (ECHO)
- My regular childcare has not been affected by the COVID-19 outbreak. (ECHO)

## Elder Care

*Some people provide regular unpaid care or assistance to a family member or friend who has a health condition, long-term illness or disability. This family member or friend could be an adult or a child. Assistance can range from a few hours of shopping and cleaning to intensive medical or personal care. Tasks can include shopping, house cleaning, cooking, giving medications, toileting assistance and so forth.*

(Source: Texas health and human services commission)

During the past month, did you provide this kind of **unpaid** care or assistance to a family member or friend?

- Yes
- No 🡪 skip to next section

How many people do you provide **unpaid** care or assistance for?

- 1
- 2
- 3 or more

*If you provide care or assistance for more than one person, please think about the person for whom you provide the* ***most*** *care and answer the following questions for that person.*

How long have you been providing care for your family member or friend?

- Less than one year
- 1 to 3 years
- 4 to 10 years
- More than 10 years

What kinds of care do you provide for your family member or friend? (mark all that apply)

- Companionship (talking, reading, keeping company) or supervision
- Transportation (driving to doctor’s appointments, driving for errands)
- Homemaking (shopping, cleaning, preparing meals)
- Personal care assistance (feeding, bathing, toileting, dressing, grooming)
- Healthcare assistance (help with medications, wound care)
- Financial assistance (paying bills, managing budget)
- None of these activities – please specify other activity: __________

How much time do you spend each week helping this friend or family member?

- 5 hours per week or less
- 6 to 20 hours per week
- 21 to 40 hours per week
- More than 40 hours per week

What is your relationship to the person for whom you provide care?

I am the person’s

- Spouse or partner
- Adult child
- Parent
- Family member
- Friend
- Other: ________________

Overall, how burdened do you feel in caring for your loved one?

- Never
- Rarely
- Sometimes
- Frequently
- Nearly always

Do you need to make work adjustments to care for your loved one (for example: taking time off or reducing work hours ?

- Yes, on a regular basis
- Yes, sometimes
- No

Do you find that caregiving is a financial strain on you?

- Yes, on a regular basis
- Yes, sometimes
- No

## Transportation

**In the following section, we will ask questions related to transportation.**

24. What was your primary mode of transportation in the past month?

- Personal vehicle
- Carpool
- Public transportation **🡪 if marked, ask 24a**
- Car rideshare services
- Bicycle (your own or shared services)
- Walking

24a. What transportation services did you use? (Mark all that apply)

- Bay Area Rapid Transit (BART)
- AC transit (buses in the east bay)
- Muni (buses and light rail in San Francisco)
- Contra Costa Transit Agency
- Ferry
- Something else, please specify: _______________________

25. In the past month, how has your experience with your primary mode of transportation changed?

- It’s been about the same **🡪 skip to next section**
- I use it more **🡪 skip to next section**
- I use it less
- I’ve switched from one mode to another

25a. What caused the change in your primary mode of transportation? (Mark all that apply)

- I have concerns about safety
- My primary mode of transportation is no longer available or is less available now
- Change in my employment situation has affected by transportation needs
- Something else, please specify: ___________________________

## Food

**The questions in this section are related to your access to food.**

26. For the next 3 statements, please indicate whether the statement is currently true, often true, sometimes true, or never true. (PBRC)

|  | **Currently true** | **Sometimes true** | **Never true** | **I don’t know** |
| --- | --- | --- | --- | --- |
| We are worried that the food we had won’t last. |  |  |  |  |
| We can’t afford to eat balanced meals. |  |  |  |  |
| We can’t get the food that me or my family want to eat. |  |  |  |  |

***If answered “currently true” or “sometimes true” to any of the statements above…***

26a. Why do you sometimes not have enough to eat (or not what you wanted to eat)? [Mark all that apply.] (CENSUS)

- Couldn’t afford to buy more food
- Couldn’t get out to buy food (for example, didn’t have transportation, or had mobility or health problems that prevented me from getting out)
- Afraid to go or didn’t want to go out to buy food
- Couldn’t get groceries or meals delivered to me
- The stores didn’t have the food I wanted

27. In the past month, how did you usually purchase groceries?

- In person at a conventional grocery store or supermarket (e.g. Target, Walmart, Safeway, Trader Joes)
- In person at a convenience store (e.g. corner market or liquor store)
- In person at a farmer’s market or other independently run food stand
- Online ordering and delivery (e.g. Instacart, Postmates)
- Some other way. Please specify: ______________________________________________

28. In the past month, have you received any reduced or free food resources (from any source, such as the government, community organizations, or individuals)? -

- Yes
- No **🡪 skip to next section**

28a. Which food resources have you received? (Mark all that apply)

- CalFresh, also known as food stamps

**🡪 if marked, ask**: Have you been able to use your CalFresh as needed? **Y/N**

- WIC (Women, Infants, and Children)

**🡪 if marked, ask**: Have you been able to use your WIC as needed? **Y/N**

- Emergency food boxes
- Free meals through the school or other programs aimed at children
- Food pantry or food bank

**🡪 if marked, ask**: Which food pantry or food bank have you received resources from?

- Home-delivered meal service like Meals on Wheels

**🡪 if marked, ask**: Which home delivered meal service have you received resources from?

- Church, synagogue, temple, mosque or other religious organization
- Shelter or soup kitchen

**🡪 if marked, ask**: Which shelter or soup kitchen have you received resources from?

- Other community program

**🡪 if marked, ask**: Which other community programs have you received resources from?

- Pandemic EBT (P-EBT)

**--> if marked, ask**: Have you been able to use your P-EBT as intended?

- Family, friends, or neighbors
- Something else. Please specify: ________________________________________

## Well-being

**In this section, we will ask some questions about your health and well-being.**

29. PROMIS 2-item global physical health, 2-item global mental health

|  |  |  |  |  |  |
| --- | --- | --- | --- | --- | --- |
| In general, how would you rate your physical health? | **Excellent** | **Very good** | **Good** | **Fair** | **Poor** |
| To what extent are you able to carry out your everyday physical activities such as walking, climbing stairs, carrying groceries, or moving a chair? | **Completely** | **Mostly** | **Moderately** | **A little** | **Not at all** |
| In general, how would you rate your mental health, including your mood and your ability to think? | **Excellent** | **Very good** | **Good** | **Fair** | **Poor** |
| In general, how would you rate your satisfaction with your social activities and relationships? | **Excellent** | **Very good** | **Good** | **Fair** | **Poor** |

30. In the last month, how often have you felt: (Perceived stress scale [PSS-4] (WIHS-MACS)

|  | **Never** | **Almost never** | **Sometimes** | **Fairly often** | **Very often** |
| --- | --- | --- | --- | --- | --- |
| That you were unable to control the important things in your life? |  |  |  |  |  |
| Confident about your ability to handle your personal problems? |  |  |  |  |  |
| That things were going your way? |  |  |  |  |  |
| Difficulties were piling up so high that you could not overcome them? |  |  |  |  |  |

31. The following is a list of concerns that some people have had since the pandemic began in March 2020. For you, what, if any, have been great sources of stress or worry for you as a result of the pandemic? (Mark all that apply) (ECHO)

- Concern for my health
- Concern for the health of family members
- Financial concerns
- Impact on work
- Impact on my child
- Impact on my community
- Impact on relationships with adult family members
- Access to food
- Access to baby supplies (e.g., formula, diapers, wipes)
- Access to personal care products or household supplies
- Access to healthcare, including mental health care
- Access to housing (including rent or mortgage responsibility)
- Ability to parent how I want or parenting responsibilities
- Ability to care for older adults or people with disabilities
- Social distancing or being quarantined
- Transportation availability and safety
- Something else. Please specify: ________________
- I am not stressed or worried about the COVID-19 outbreak

32. In your day-to-day life, how often do any of the following things happen to you? (Everyday discrimination scale)

|  | **Almost everyday** | **At least once a week** | **A few times a month** | **A few times a year** | **Less than once a year** | **Never** |
| --- | --- | --- | --- | --- | --- | --- |
| You are treated with less courtesy than other people are |  |  |  |  |  |  |
| You are treated with less respect than other people are |  |  |  |  |  |  |
| You receive poorer service than other people at restaurants or stores |  |  |  |  |  |  |
| People act as if they think you are not smart |  |  |  |  |  |  |
| People act as if they are afraid of you |  |  |  |  |  |  |
| People act as if they think you are dishonest |  |  |  |  |  |  |
| People act as if they’re better than you are |  |  |  |  |  |  |
| You are called names or insulted |  |  |  |  |  |  |
| You are threatened or harassed |  |  |  |  |  |  |

***If answered “A few times a year” or more frequently to at least one question, ask*:**

32a. What do you think is the main reason for these experiences? (Mark all that apply).

- Your ancestry or national origins
- Your gender
- Your race/ethnicity
- Your age
- Your religion
- Your height, weight, or some other aspect of your physical appearance
- Your sexual orientation
- Your education or income level

**NEW SECTION – COPING**

Which of the following strategies [have been/were] helpful to YOU during the COVID-19 outbreak?

|  | Not at all / I didn’t try this | Slightly | Somewhat | Moderately | Very much |
| --- | --- | --- | --- | --- | --- |
| Texting, calling or video-calling family members or friends |  |  |  |  |  |
| Talking to therapist |  |  |  |  |  |
| Exercising/walking |  |  |  |  |  |
| Cooking/baking |  |  |  |  |  |
| Arts and crafts project |  |  |  |  |  |
| Cleaning/doing household chores |  |  |  |  |  |
| Playing games with family members |  |  |  |  |  |
| Playing online games |  |  |  |  |  |
| Virtual entertainment (concerts, live shows, sports, museums, etc.) |  |  |  |  |  |
| Reading |  |  |  |  |  |
| Using social media |  |  |  |  |  |
| Other: (specify) |  |  |  |  |  |

**NEW SECTION – RESILIENCE**

Please respond to each item by marking one box per row.

|  | Strongly disagree | Disagree | Neutral | Agree | Strongly agree |
| --- | --- | --- | --- | --- | --- |
| I tend to bounce back quickly after hard times. |  |  |  |  |  |
| I have a hard time making it through stressful events |  |  |  |  |  |
| It does not take me long to recover from a stressful event. |  |  |  |  |  |
| It is hard for me to snap back when something bad happens. |  |  |  |  |  |
| I usually come through difficult times with little trouble. |  |  |  |  |  |
| I tend to take a long time to get over set-backs in my life. |  |  |  |  |  |

## Social support

People sometimes look to others for companionship, assistance, or other types of support. These questions are about how often you have had this type of support in general

| **In the past 12 months, how often is each of the following kinds of support available to you if you need it? Choose one number from each line.** | None of the time | A little of the time | Some of the time | Most of the time | All of the time |
| --- | --- | --- | --- | --- | --- |
| 1. Someone you can count on to listen to you when you need to talk |  |  |  |  |  |
| 1. Someone to give you information to help you understand a situation |  |  |  |  |  |
| 1. Someone to give you good advice about a crisis |  |  |  |  |  |
| 1. Someone to confide in or talk to about yourself or your problems |  |  |  |  |  |
| 1. Someone whose advice you really want |  |  |  |  |  |
| 1. Someone to share your most private worries and fears with |  |  |  |  |  |
| 1. Someone to turn to for suggestions about how to deal with a personal problem |  |  |  |  |  |
| 1. Someone who understands your problems |  |  |  |  |  |
| 1. Someone to help you if you were confined to bed |  |  |  |  |  |
| 1. Someone to take you to the doctor if you needed it |  |  |  |  |  |
| 1. Someone to prepare your meals if you were unable to do it yourself |  |  |  |  |  |
| 1. Someone to help with daily chores if you were sick |  |  |  |  |  |
| 1. Someone who shows you love and affection |  |  |  |  |  |
| 1. Someone to love and make you feel wanted |  |  |  |  |  |
| 1. Someone who hugs you |  |  |  |  |  |
| 1. Someone to have a good time with |  |  |  |  |  |
| 1. Someone to get together with for relaxation |  |  |  |  |  |
| 1. Someone to do something enjoyable with |  |  |  |  |  |
| 1. Someone to do things with to help you get your mind off things |  |  |  |  |  |

## COVID-19

**The questions in this section are related to your household’s experiences with COVID-19 illness.**

35. In the past two month, have you or someone in your household tested positive for coronavirus?

- Yes
- No

34. In the past two month, have you or someone in your household had symptoms of COVID-19 (since March 2020)? Common symptoms of COVID-19 include newly developed fever, dry cough, and general feelings of “tiredness”. Less common symptoms include aches and pains, sore throat, diarrhea, conjunctivitis, headache, loss of taste or smell, or rashes on skin/discoloration of fingers and toes.

- Yes
- No 🡪 Skip to 39

36. In the past month, have you or someone in your household been hospitalized with COVID?

- Yes
- No

37. In the past month, have you or someone in your household been in the ICU (intensive care unit) with COVID?

- Yes
- No

38. In the past month, has someone in your household died of COVID?

- Yes
- No

**39. Have you received the COVID-19 vaccine? (Mindy – not sure if important to specify # of doses/which vaccine; I.e., have you received at least one dose of the Pfizer or Moderna vaccine or the Johnson and Johnson vaccine?)**

- Yes --> Skip to question 41
- No

**40. Do you plan to receive the COVID-19 vaccine?**

- Yes
- No

**41. With whom/where did you receive your COVID-19 vaccine? __________________________**

## Residence

In the past two months, did your address change?

- Yes
- No

**Only ask again if answer to whether housing changes is ‘Yes’.**

**Address of residence**

This information will be used to inform organizations where resources are needed, or where useful and effective resources are available during the pandemic. Your address will be treated confidentially.

41. What you your usual address of residence?

Street number and street ________________________________

City ____________________________________

Zip code ____________________________________

If you do not want to or are not able to provide your usual address of residence, can you share the nearest cross-streets to your usual address of residence?

Street #1

Street #2

City
